# Unexpected costs: the impact of Long-Term Care Insurance on housing prices

**DOI:** 10.3389/fpubh.2025.1702221

**Published:** 2025-11-13

**Authors:** Xin Yang, Jing Wen, Weijie Du, Zhiding Hu

**Affiliations:** 1 School of Geographical Sciences, East China Normal University, Shanghai, China; 2 Zhejiang Economic Information Center, Hangzhou, China

**Keywords:** Long-Term Care Insurance, housing prices, older adults migration, difference-in-differences, aging policies

## Abstract

**Introduction:**

China’s accelerated demographic aging has intensified scholarly interest in the institutional design and socio-economic impacts of the Long-Term Care Insurance (LTCI) pilot program. While its social and healthcare effects have been widely examined, little is known about its broader economic implications, particularly its impact on regional housing markets.

**Methods:**

Using panel data for 285 Chinese cities from 2003 to 2022, this study employs a staggered difference-in-differences (DID) approach to estimate the causal effect of LTCI on housing prices. To ensure robustness, a series of additional tests are conducted, including propensity score matching (PSM), Bacon decomposition, system GMM estimation, placebo tests, and model averaging.

**Results:**

The findings indicate that the implementation of LTCI significantly increases housing prices. The migration of older adults into pilot cities serves as a key mechanism through which LTCI affects housing prices, as improved healthcare accessibility and enhanced living environments attract older adults and stimulate local housing demand. Heterogeneity analysis reveals that the effect is more pronounced in eastern regions, in areas with better environmental quality, and in cities offering home-based care services, while no significant differences are observed between pilot types.

**Discussion:**

These results underscore the secondary economic impacts of long-term care policies. By linking LTCI to the migration of older adults and housing demand, this study deepens understanding of how welfare policies interact with housing markets in aging societies.

## 1 Introduction

Housing prices have become a critical factor shaping residents’ quality of life, social stability, and economic development (1). Driven by ongoing urbanization and structural economic transformation, China has experienced a rapid rise in housing prices, intensifying household financial burdens and triggering a range of societal challenges, including widening income inequality, imbalanced migration patterns, and reduced household consumption capacity (2). Meanwhile, against the backdrop of rapid population aging, the housing and care needs of the older adults have become increasingly prominent, making their effective provision a pressing public concern (3). To address the escalating care burden, the Chinese government launched the Long-Term Care Insurance (LTCI) pilot program in 2016 (4). This policy provides essential daily living assistance and medical services to individuals with long-term disabilities, thereby easing pressures on both families and society as a whole (5). While the program has yielded notable social benefits during its pilot phase, it has also generated unintended economic consequences, one of which is its potential spillover effect on regional housing prices.

While previous studies have investigated the health consequences of LTCI and its effects on healthcare expenditures (6), the economic implications of its interaction with the housing market remain insufficiently explored (7–9). In other words, important questions regarding how the implementation of LTCI affects regional housing prices, the mechanisms through which these effects operate, and whether different policy designs generate differential effects on housing prices, remain underexplored. Notably, the implementation of LTCI, as a public pension policy, may attract older adults into pilot regions through improved care provision, or alter household housing and consumption decisions, thereby potentially affecting local housing demand and prices.

Given the central role of the housing market in household wealth accumulation and the allocation of social resources (10), examining the impact of LTCI on regional housing prices and its underlying mechanisms is essential. Such analysis not only deepens our understanding of the secondary economic effects of public policy but also informs the optimization of policy design to balance social welfare with market efficiency. Furthermore, clarifying how LTCI influences the migration patterns of older adults and regional housing demand can provide empirical evidence and theoretical insights for government decision-making in regional development planning and the allocation of long-term care resources, thereby advancing the modernization of governance in an aging society.

This study uses a panel dataset of 285 cities from 2003 to 2022 to evaluate the impact of LTCI on regional housing prices in China through the staggered difference-in-differences (DID) model. The results show that LTCI raises regional housing prices, and this finding remains robust after a series of checks, including propensity score matching (PSM), heterogeneity-robust estimators, Bacon decomposition, system generalized method of moments (GMM), placebo tests, and model averaging. Further analysis indicates that the main mechanism is the in-migration of older adults into LTCI cities, which increases local housing demand rather than real estate investment. In addition, the agglomeration of medical resources and improvements in the living environment serve as key factors attracting older adults to LTCI cities. Finally, heterogeneity analysis by policy design and regional context shows that the spillover effect of LTCI is stronger in cities offering home-based LTCI services, in eastern regions, and in areas with lower pollution levels. In contrast, no significant differences are observed across pilot types, suggesting that the welfare effects of LTCI are largely independent of the implementing agency.

This study makes several key contributions. First, to the best of our knowledge, this is the first study to investigate the influence of LTCI on housing prices, thereby extending the literature on its economic implications beyond health-related effects. Most existing studies focus on outcomes such as individual health (11), healthcare expenditure (6), and household welfare (12), while neglecting the housing market as a channel for welfare transmission. Our results show that LTCI pilot programs increased regional housing prices by 8.1–8.6%, equivalent to 588–624 RMB per square meter, quantifying an additional economic burden and providing evidence relevant to balancing welfare and market efficiency.

Second, this study deepens the understanding of how LTCI influences population mobility and, consequently, housing market dynamics. Previous research has mainly examined the role of LTCI in the out-migration of older adults (13), but we show that it also promotes in-migration by improving healthcare accessibility and environmental amenities in pilot cities. This inflow of older adults increases housing demand and raises regional housing prices. At the same time, the findings clarify that this effect is demand-driven rather than investment-induced, as LTCI does not stimulate real estate investment despite reducing medical and non-medical expenditures (14).

Third, the findings contribute to the understanding of older adult migration, highlighting its transformation from a market-oriented behavior to a policy-induced spatial process. Previous studies link healthcare resources (15) and environmental quality (16) to older adults migration, but mainly in market-driven or preference-oriented contexts. Our analysis indicates that LTCI increases healthcare resources and green spaces in pilot cities, which in turn attract older adults. Unlike Sumita et al. (13), which focused on out-migration, we highlight the role of LTCI in in-migration and its implications for resource allocation and population mobility in an aging society.

The remainder of the study proceeds as follows. Section 2 introduces the policy background of LTCI. Section 3 discusses prior research on LTCI and the housing market and articulates the hypotheses. Section 4 outlines the data construction and empirical design. Section 5 reports the core results together with robustness examinations. Section 6 extends the analysis to mechanisms and heterogeneity. Section 7 presents a discussion of the empirical findings and related policy implications, while Section 8 summarizes the main conclusions of the study.

## 2 Policy regime

In 2016, China’s Ministry of Human Resources and Social Security issued policy documents to launch the pilot program of LTCI, after which related policies and debates on long-term care have continued to evolve and deepen (4, 17). One core institutional feature of LTCI is its reliance on participants’ enrollment in basic medical insurance schemes, which reflects its integration into the broader health security system. Building on this shared institutional foundation, local pilot programs have developed with broadly similar policy goals, but still exhibit notable differences in implementation. While the overall design of LTCI policies is broadly similar across pilot areas, several notable differences persist in four dimensions [see Supplementary Table 1, verified against existing studies such as Ai et al. (17)].

First, in terms of pilot type, the Ministry of Human Resources and Social Security announced two batches of nationally designated LTCI pilots in 2016 and 2020 (hereafter referred to as designated pilots). However, in some areas with strong social welfare demand and sufficient fiscal capacity, local governments launched LTCI pilots independently (hereafter referred to as self-initiated pilots). For example, Qingdao in Shandong Province began implementing LTCI in urban areas as early as 2012. Self-initiated pilots account for approximately 38% of pilot cities. Second, regarding service coverage, roughly 10% of pilot areas (e.g., Ningbo) reimburse only institutional care, whereas the majority cover both institutional and home-based care. Third, in terms of reimbursement methods, around 77% of pilots cover only in-kind services provided by care institutions, while 23% adopt a mixed approach that combines service reimbursement with cash payments to beneficiaries and their families. Finally, considering coverage eligibility, roughly 54% of pilot sites restrict benefits to members of the urban employee medical insurance scheme, whereas the others expand reimbursement to encompass participants in the urban–rural resident scheme. This indicates that in some pilot cities, only urban employee participants are eligible for LTCI benefits, which institutionally limits the extent to which older adult migrants, particularly those without urban employee status, can directly benefit from the policy. Nevertheless, by enhancing the allocation of older adult care resources and expanding service availability, the policy can influence the migration decisions of older adults even when formal eligibility is limited. In light of this possibility, we provide a transitional explanation to guide the mechanism analysis in Section 6.1.1, which focuses on healthcare resources and living environment. Although the baseline model does not directly control for policy design differences, its goal is to estimate the average treatment effect across all pilots. We then explicitly account for variation in pilot type and service coverage in the heterogeneity analysis presented in Section 6.2.

## 3 Theoretical context and hypothesis development

### 3.1 Health effects of Long-Term Care Insurance

Prior studies have assessed the health implications of LTCI from diverse perspectives. Empirical results based on micro-level longitudinal data from Chengdu indicate that the adoption of LTCI significantly mitigates mortality risks among older adults and enhances longevity (18). This finding is supported by evidence from Korea, where Kim and Mitra report positive effects of LTCI on the health status of older adults (7). Regarding mental health, LTCI mitigates depressive symptoms by enhancing daily companionship and social interaction, with particularly pronounced effects among older adults with chronic diseases or disabilities and those residing in less-developed regions (19). In terms of healthcare utilization, LTCI substitutes for part of the demand for outpatient services through home care subsidies and in-home services, thereby reducing the frequency and duration of hospital stays and lowering medical expenditures (20). In addition, the health effects of LTCI extend beyond insured individuals, generating welfare spillovers for family members (21).

From the perspective of welfare economics, these health improvements translate into substantial social welfare gains (22). LTCI functions as a quasi-public good, providing risk pooling and redistributive benefits that cannot be achieved by individual markets alone. By reducing uncertainty associated with disability and long-term care needs, LTCI increases the expected utility of consumption for older households and alleviates welfare losses arising from catastrophic health expenditures (19, 23). Moreover, by substituting private caregiving costs with publicly financed services, LTCI enhances allocative efficiency and reduces inequality in access to healthcare resources. Consequently, the observed health benefits of LTCI do not merely reflect improvements in individual well-being but also signify a broader welfare-enhancing effect at the societal level, forming the economic rationale for the policy’s continued expansion and institutionalization.

Although existing research has developed a theoretical framework for understanding the health effects of LTCI, an important concern is that welfare disparities between pilot and non-pilot regions may induce institutional migration of older adults from non-pilot to pilot areas. Such migration could impose additional economic burdens and challenge the long-term sustainability of the system. Therefore, assessing the potential economic costs associated with LTCI implementation is essential for achieving a balanced approach between welfare provision and fiscal responsibility.

### 3.2 Spillover effects of healthcare accessibility on housing prices

With the progress of urbanization, the accessibility of healthcare resources has become an important determinant of regional housing prices. Numerous empirical studies show that the proximity of hospitals and other healthcare facilities directly affects nearby housing prices. With increasing demand for medical services, residences located near hospitals often command a price premium—an effect that is particularly pronounced in aging societies where access to healthcare is especially critical (24, 25). A study in Taipei found that housing prices are generally higher near hospitals because healthcare accessibility, as a key community amenity, can significantly enhance surrounding property values (26). Mainland Chinese evidence substantiates this point, indicating that improved hospital accessibility raises housing prices, as households value proximity to healthcare, thereby illustrating the price-enhancing role of medical resources (24, 27).

Adopting a spatial population economics approach, differences in healthcare accessibility not only shape regional welfare levels but also influence the spatial allocation of population (28). Cities with more abundant and higher-quality medical resources provide greater expected utility for residents, particularly for older adult households that are more sensitive to healthcare availability (29). As individuals seek to maximize welfare subject to income and housing constraints, these regional disparities in public service provision induce selective migration flows toward cities with superior healthcare infrastructure (30). Over time, this process gives rise to population sorting, in which older or health-conscious groups cluster in welfare-advantaged regions, thereby intensifying demographic concentration and housing demand (31). Consequently, the unequal spatial distribution of healthcare services functions as both a determinant of population mobility and a catalyst for housing market differentiation across regions.

Amid China’s demographic transition, long-term care policy initiatives and service accessibility are likely to emerge as crucial determinants of housing market dynamics. By providing a more convenient living environment for older adults, such policies can act as a form of health-related resource that drives housing prices upward. However, the possible effects of long-term care policies on regional housing values remain underexplored in the literature. Investigating the relationship between older adult care policies, such as LTCI, and housing price premiums can help bridge this gap and provide a novel perspective on the interaction between public policy and the housing market.

### 3.3 Long-Term Care Insurance and regional housing prices

Based on the above theoretical considerations, LTCI may generate not only health benefits but also spillover effects on regional housing markets. Specifically, the implementation of LTCI can reshape the spatial equilibrium of welfare and housing markets through a welfare–migration–housing mechanism. This may mainly be achieved through two mechanisms: population migration and household consumption.

First, in terms of population migration, LTCI improves regional healthcare infrastructure and service accessibility, thereby enhancing the overall level of public welfare. These institutional improvements create spatial welfare differentials between pilot and non-pilot cities, offering a policy-induced incentive for older adults to relocate in pursuit of better long-term care services and living conditions. By offering more comprehensive care at lower costs (7), LTCI creates a welfare advantage for pilot cities relative to non-pilot areas. This disparity attracts the in-migration of older adults (13), increasing local housing demand, especially in regions where LTCI coverage is more generous or accessible, and ultimately raising regional housing prices (32).

Second, in terms of household consumption, LTCI reduces out-of-pocket medical expenses (6, 33), thereby improving household liquidity and lowering the need for precautionary savings against future care risks. The resulting increase in disposable resources can stimulate other forms of consumption, including real estate investment or housing upgrades, which in turn contributes to higher regional housing prices (14, 34), This property consumption channel further amplifies the housing price effect of LTCI. Accordingly, we propose the following hypotheses:

*H1*: LTCI increases regional housing prices.

*H2*: LTCI raises regional housing prices by improving welfare provision and stimulating the migration of older adults.

*H3*: LTCI raises regional housing prices by stimulating real estate investment.

## 4 Research design

### 4.1 Data

**Figure 1:**
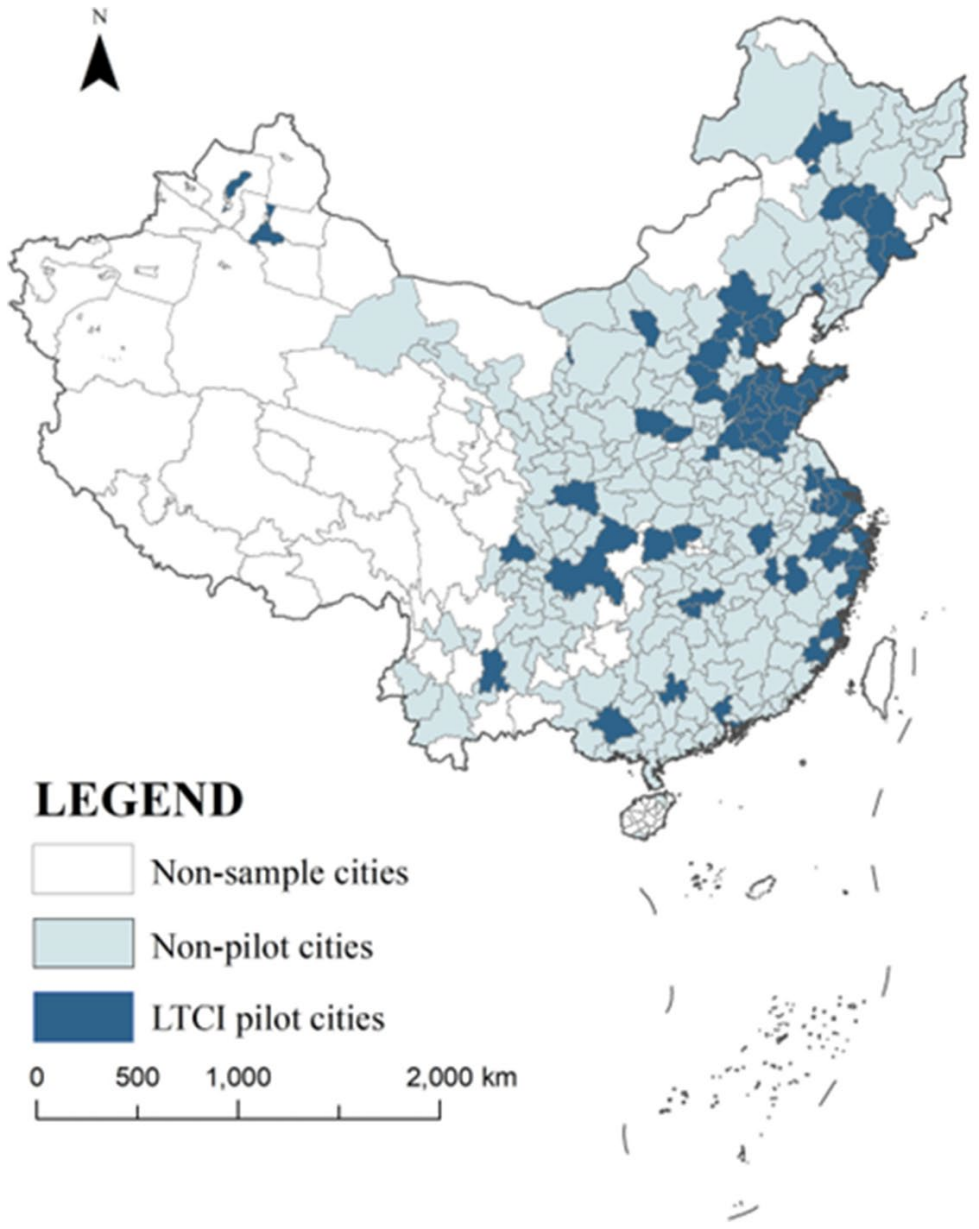
Research sample diagram.

The study utilizes panel data for 285 Chinese cities from 2003 to 2022, including 69 LTCI pilot cities and 216 non-pilot cities. Although 76 cities were officially designated as LTCI pilots, 7 of them were excluded from the sample due to missing data in key variables. Figure 1 illustrates the spatial distribution of the final sample. Seven pilot cities were excluded from the sample due to missing key data. Housing price data are sourced from Anjuke^1^, which has over 25 million monthly active users as of 2024 and provides both monthly and annual city-level housing price information across China (35). The platform provides detailed records for most residential communities nationwide, including geographic coordinates, total number of units, property management companies, property fees, and historical price trends, offering reliable support for this research.

Data on LTCI are sourced from the official website of the Ministry of Human Resources and Social Security of China^2^. Since some non-pilot cities implemented LTCI independently and some pilot cities delayed implementation, additional information was manually collected and cross-validated with existing studies (17, 36).

Data on older adult migration are obtained from Huang et al. (37). Other prefecture-level indicators are drawn from the China City Statistical Yearbook, the China Statistical Yearbook for Regional Economy, and the China Statistical Yearbook. All price variables are deflated to 2003 constant prices.

### 4.2 Variable definitions

#### 4.2.1 Dependent variable

The dependent variable of this study is the annual average housing price of each city, expressed in natural logarithms.

#### 4.2.2 Independent variable

The key independent variable is an interaction term between a dummy variable indicating whether a city is an LTCI pilot city (*Treat_i_*) and a dummy variable indicating whether the time is after the policy implementation (*Post_t_*). The interaction term (*LTCI_i, t_*) equals 1 if city *i* implemented LTCI in year *t*, and 0 otherwise.

#### 4.2.3 Other variables

In addition to the core variables above, several confounding and mediating factors are considered that may influence the relationship between LTCI and regional housing prices. Table 1 presents the definitions for all variables used in this study.

**Table 1:** Variable definitions.

Variable	Definition
Dependent variable	
House_price	Natural log of regional housing prices, with data obtained from Anjuke.
Independent variable	
LTCI	Equals 1 if city *i* implemented LTCI in year *t*, and 0 otherwise.
Mediating variables	
Migration_in	Inflow of older adult migrants. Due to the limited availability of data on older adult migration, following existing research, estimates are derived from the 6th and 7th National Population Censuses (2010, 2020) and the 1% National Population Sample Survey conducted in 2005 and 2015, aggregated at the provincial level over five-year intervals.
Migration_out	Outflow of older adult migrants.
Net_migration	Net inflow of older adults.
lnRE_Completions	Natural log of completed real estate investment.
lnRE_Practitioners	Natural log of the number of employees in the real estate sector.
lndoctor	Natural log of the number of licensed or assistant doctors.
lnhospital	Natural log of the number of hospitals.
Greenpark	Area of urban parks and green spaces measured in 10,000 hectares.
Control variables	
indus	Share of secondary industry in GDP.
edu	Ratio of education expenditure to GDP.
fis	Ratio of general budgetary fiscal expenditure to GDP.
fin	Ratio of the loans from financial institutions to GDP.
sci	Ratio of science expenditure to GDP.
so_2_	Ratio of industrial SO_2_ emissions to GDP.
lnpop	Natural log of registered population.
lnperGDP	Natural log of GDP per capita.

### 4.3 Model specifications

The DID method compares outcomes across two time periods (before and after policy implementation) and two groups (treatment and control). The policy effect is identified from differences both over time within groups and between groups at a given point in time. Incorporating two-way fixed effects into the DID framework further controls for unobserved heterogeneity across regions and years, thereby enhancing the robustness and precision of the estimated policy effect.

Because LTCI was introduced in multiple pilot batches, we adopt a staggered DID identification strategy. By comparing changes in housing prices between LTCI pilot cities and non-pilot cities before and after implementation, we estimate the impact of LTCI on regional housing prices. Specifically, officially designated LTCI pilot cities are assigned to the treatment group, while all other cities are assigned to the control group. The baseline estimation model is specified as follows:


$$\text{House}\_\text{pric}e_{i,t}=\beta_{0}+\beta_{1}\mathrm{LTC}I_{i,t}+\beta_{2}\text{Contro}l_{i,t}+\delta_{i}+\gamma_{t}+\varepsilon_{i,t}$$ (1)

where *i* and *t* denote city and year, respectively. $\text{House}\_\text{pric}e_{i,t}$ is the natural log of regional housing prices. $\mathrm{LTC}I_{i,t}$ is a dummy variable equal to 1 if city *i* is affected by the LTCI policy in year *t*. $\text{Contro}l_{i,t}$ is a vector of control variables. $\delta_{i}$ represents city fixed effects, and $\gamma_{t}$ represents year fixed effects. $\varepsilon_{i,t}$ is the error term.

To investigate the underlying mechanisms, we employ the following estimation model:


$$M_{i,t}=\beta_{0}+\beta_{1}\mathrm{LTC}I_{i,t}+\beta_{2}\text{Contro}l_{i,t}+\delta_{i}+\gamma_{t}+\varepsilon_{i,t}$$ (2)

where $M_{\mathrm{it}}$ denotes the mediator variable, the in-migration of older adults, real estate investment, and factors derived from in-migration including the healthcare resource and the living environment. All other specifications follow Equation 1.

### 4.4 Data summary

Table 2 summarizes the descriptive statistics for the primary variables. The data characteristics are broadly consistent with those reported in related studies, indicating their reliability.

**Table 2:** Descriptive statistics of variables.

Variables	(1)	(2)	(3)	(4)	(5)
	Observations	Mean	S. D.	Minimum	Maximum
House_price	5,024	8.334	0.638	6.658	11.008
LTCI	5,024	0.054	0.225	0	1
indus	5,024	0.482	0.125	0.086	0.910
edu	5,024	0.024	0.013	0.001	0.167
fis	5,024	0.498	0.477	0.033	6.798
fin	5,024	2.320	2.135	0.075	84.661
sci	5,024	0.005	0.006	0	0.072
so_2_	5,024	2.214	4.308	0	90.251
lnpop	5,024	4.625	0.779	2.645	7.820
lnperGDP	5,024	10.571	0.814	−1.763	12.993
Migration_in	4,030	5.626	7.848	0.170	41.120
Migration_out	4,030	6.612	4.570	0.210	23.880
Net_migration	4,030	−0.986	8.662	−22.660	30.800
lnRE_Completions	4,388	13.420	1.581	−0.580	17.609
lnRE_Practitioners	4,480	8.142	1.328	0	13.069
lndoctor	5,014	8.002	0.944	4.719	11.659
lnhospital	5,020	4.992	0.755	1.609	8.024
Greenpark	4,999	0.148	0.288	0	3.690

The dataset is an unbalanced panel due to missing values in certain control variables for some cities and years; incomplete records were excluded to maintain data consistency.

## 5 Empirical results

### 5.1 Benchmark results

Empirical estimates of LTCI’s effect on regional housing prices are provided in Table 3. Across Columns (1) and (2)—excluding and including controls—the coefficients are positive and statistically significant at the 1% level. The results indicate that the introduction of LTCI increased regional housing prices by approximately 8.1 to 8.6%. Using the untransformed data, where the mean housing price in the treatment group is RMB 7,257.24 per square meter (USD 1010.76 per square meter, based on an exchange rate of 7.18 RMB per USD), this effect translates into an increase of roughly RMB 587.84 to RMB 624.12 per square meter (USD 81.87 to USD 86.93 per square meter).

**Table 3:** Baseline regression results: impact of LTCI on housing prices.

Variables	House_price				
	TWFE		PSM-DID		
	(1)	(2)	(3)	(4)	(5)
LTCI	0.081*** (0.025)	0.086*** (0.024)	0.072*** (0.022)	0.088*** (0.023)	0.088*** (0.023)
Control	No	Yes	Yes	Yes	Yes
City FE	Yes	Yes	Yes	Yes	Yes
Year FE	Yes	Yes	Yes	Yes	Yes
Observations	5,024	5,024	3,243	4,888	4,888
R-squared	0.942	0.945	0.945	0.945	0.945

***, **, and * denote statistical significance at the 1, 5, and 10% levels, respectively. Standard errors clustered at the city level are reported in parentheses. Column (3) applies 1:4 nearest neighbor matching, Column (4) applies caliper matching, and Column (5) applies kernel matching. Results for control variables are omitted for brevity.

The Fifth National Survey on the Living Conditions of the Older Adults in Urban and Rural China (2024) reports that older adults have an average housing area of 121.7 square meters. Based on this estimate, the additional housing cost attributable to LTCI is approximately RMB 70,656.50–75,955.74 (USD 9,840.74–10,578.79) per older adults.

There remains the possibility that treatment and control groups differ systematically, which could bias the estimates. LTCI pilots are more likely to be introduced in relatively affluent cities, characterized by higher household incomes and correspondingly higher housing prices. Such bias could affect the reliability of the empirical estimates. To address this issue, we employ the PSM-DID approach. Columns (3)–(5) of Table 3 present the results, and the corresponding balance tests are reported in Supplementary Tables 2–4 and Supplementary Figures 1–3. The persistence of a positive LTCI effect on housing prices, despite adjustments for selection bias, lends empirical support to Hypothesis H1.

### 5.2 Robustness checks

#### 5.2.1 Parallel trends test

**Figure 2:**
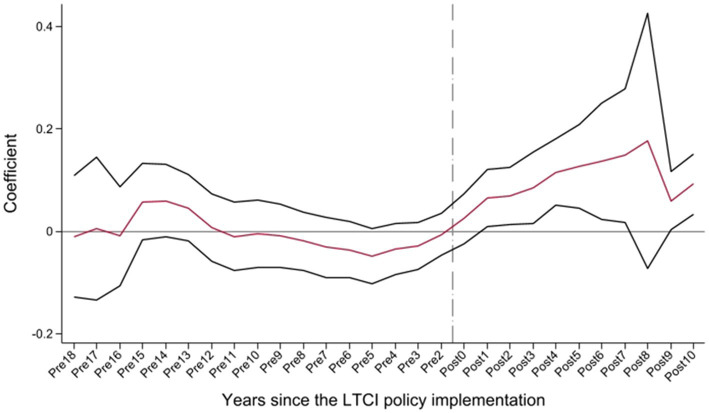
Parallel trends.

To ensure the internal validity of the DID design, we first examine whether the pilot and non-pilot cities followed similar pre-policy trends in housing prices. Since DID estimation hinges on the validity of the parallel-trends assumption, we verify this assumption using an event-study approach (Figure 2). The regression estimates for regional housing prices indicate that, in the pre-policy period (Pre18-Pre2), the 95% confidence intervals consistently include 0, suggesting that housing prices in pilot and non-pilot cities followed similar trends prior to the implementation of LTCI. By contrast, during the post-policy period (Post0–Post10), the 95% confidence intervals are largely positive and statistically different from zero, indicating a significant increase in housing prices in pilot cities. These findings support the interpretation of the baseline estimates as reflecting the causal effect of LTCI. The parallel-trends test thus confirms that the DID framework is valid and that the observed price increase can be causally attributed to LTCI implementation.

#### 5.2.2 Parallel trends test with heterogeneity-robust estimators

**Figure 3:**
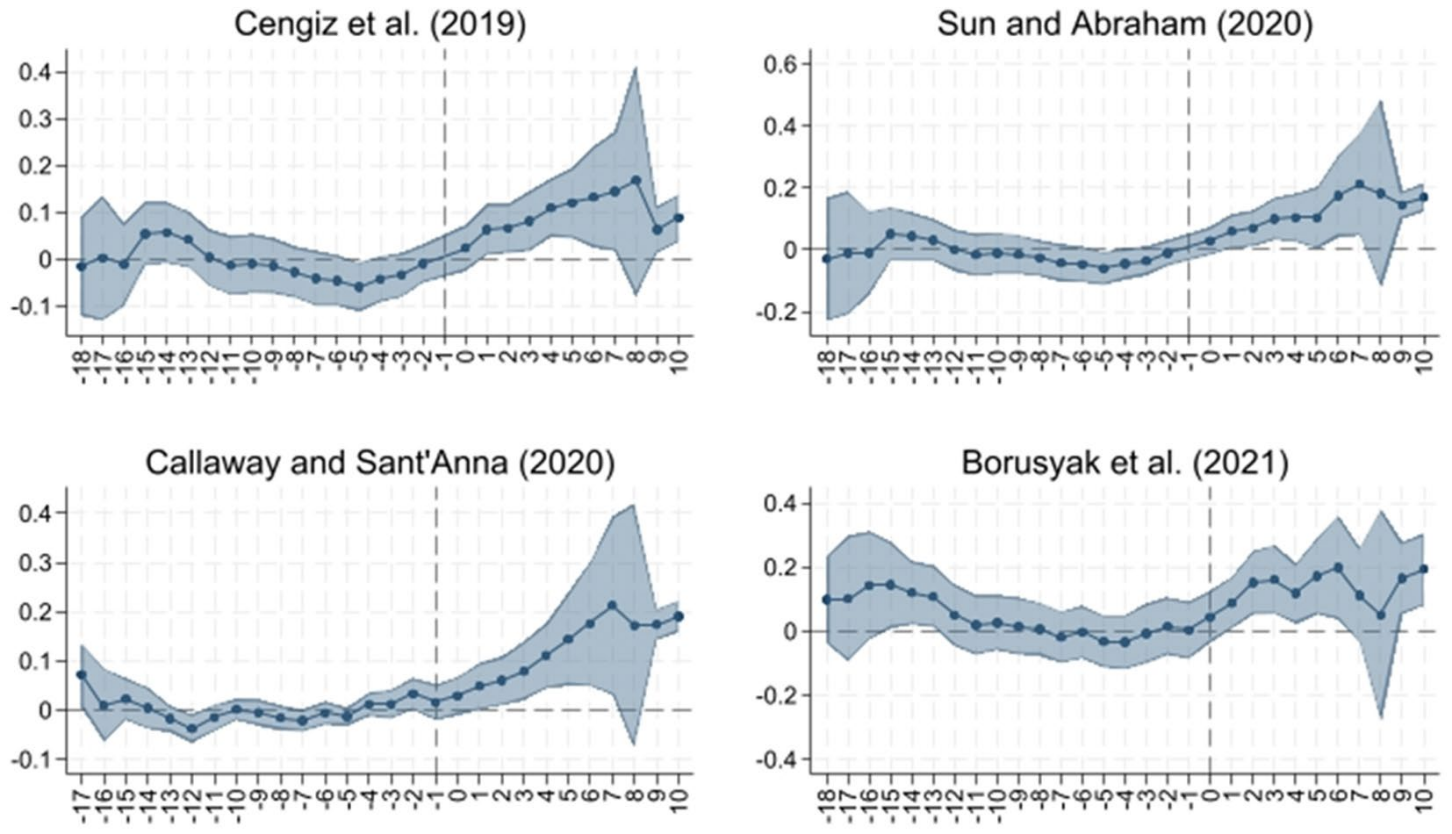
Parallel trend analysis using heterogeneity-robust estimation.

To account for potential bias from heterogeneous treatment effects, we test whether the estimated impact of LTCI remains robust when relaxing the homogeneity assumption of the TWFE-DID model. The TWFE-DID approach assumes homogeneous treatment effects across units and over time. In reality, this assumption is often violated because treatment effects may differ by group, policy timing, or treatment intensity. This heterogeneity may bias TWFE-DID estimates. To mitigate this concern, we adopt recent methodological advances and re-estimate the parallel trends test using heterogeneity-robust estimators (38–41). Evidence from Figure 3 shows that the effect of LTCI on regional housing prices is persistently significant, reinforcing the causal interpretation.

#### 5.2.3 Bacon decomposition

The LTCI policy was implemented in two pilot phases, resulting in variation in treatment timing across cities. Although the baseline analysis employs a staggered DID model to account for this variation, differences in treatment timing may still introduce bias (42). To mitigate this concern, we apply Goodman-Bacon’s (43) decomposition technique, which disaggregates the DID estimator into its underlying comparisons. Results are summarized in Table 4 and illustrated in Supplementary Figure 4.

**Table 4:** Goodman-Bacon decomposition results.

Panel A		Panel B	Coefficient β	Weight
Coefficient	0.0860***	Timing_groups	0.0295	0.0761
S. D.	0.0240			
Z-statistic	3.59	Never_v_timing	0.0891	0.8996
*p*-value	0.000			
95% CI	(0.042, 0.151)	Within	0.1716	0.0243

***, **, and * indicate statistical significance at the 1, 5, and 10% levels, respectively.

Panel A of Table 4 shows that the estimated coefficient remains positive and statistically significant at the 1% level, consistent with the baseline results. Panel B reports the decomposition weights and average effects for each type of comparison: later-treated cities compared with earlier-treated cities (Timing_groups), changes within the same group over time (Within), and later-treated cities compared with cities that were never treated (Never_v_timing). The first two types account for only 7.61 and 2.43% of the total weight, respectively, whereas the last type contributes the majority of the weight (89.96%) and explains 93.20% of the overall coefficient (0.8996 × 0.0891÷0.0860). These findings confirm that the staggered DID model is an appropriate framework for estimating the impact of LTCI on housing prices.

#### 5.2.4 System GMM

Regional housing prices partly reflect local economic development and household income levels. Consequently, the central government may have a tendency to select pilot cities for the LTCI program based on these characteristics, which could introduce potential reverse causality and bias the results of this study. We employ the system GMM estimator to further mitigate endogeneity issues. The results are reported in Table 5. The AR (1) test confirms the presence of first-order serial correlation, whereas the AR (2) test provides no evidence of second-order correlation. Moreover, the Hansen test does not reject the null of instrument validity, suggesting that the instruments are appropriately specified. Overall, the results confirm that the model is well specified and that the main findings remain robust. These results further reinforce the conclusion that LTCI implementation exerts a significant and robust positive effect on regional housing prices.

**Table 5:** System GMM estimation results.

Variables	One-step		Two-step	
	Standard	Orthogonal	Standard	Orthogonal
	(1)	(2)	(3)	(4)
LTCI	0.2412*** (0.092)	0.2393*** (0.088)	0.1982*** (0.069)	0.2094*** (0.064)
Control	Yes	Yes	Yes	Yes
City FE	Yes	Yes	Yes	Yes
Year FE	Yes	Yes	Yes	Yes
Observations	4,719	4,719	4,719	4,719
AR (1)	0.000	0.000	0.000	0.000
AR (2)	0.103	0.119	0.111	0.374
Hansen test	0.210	0.146	0.257	0.146

***, **, and * denote statistical significance at the 1, 5, and 10% levels, respectively. Standard errors clustered at the city level are reported in parentheses.

#### 5.2.5 Placebo test

**Figure 4:**
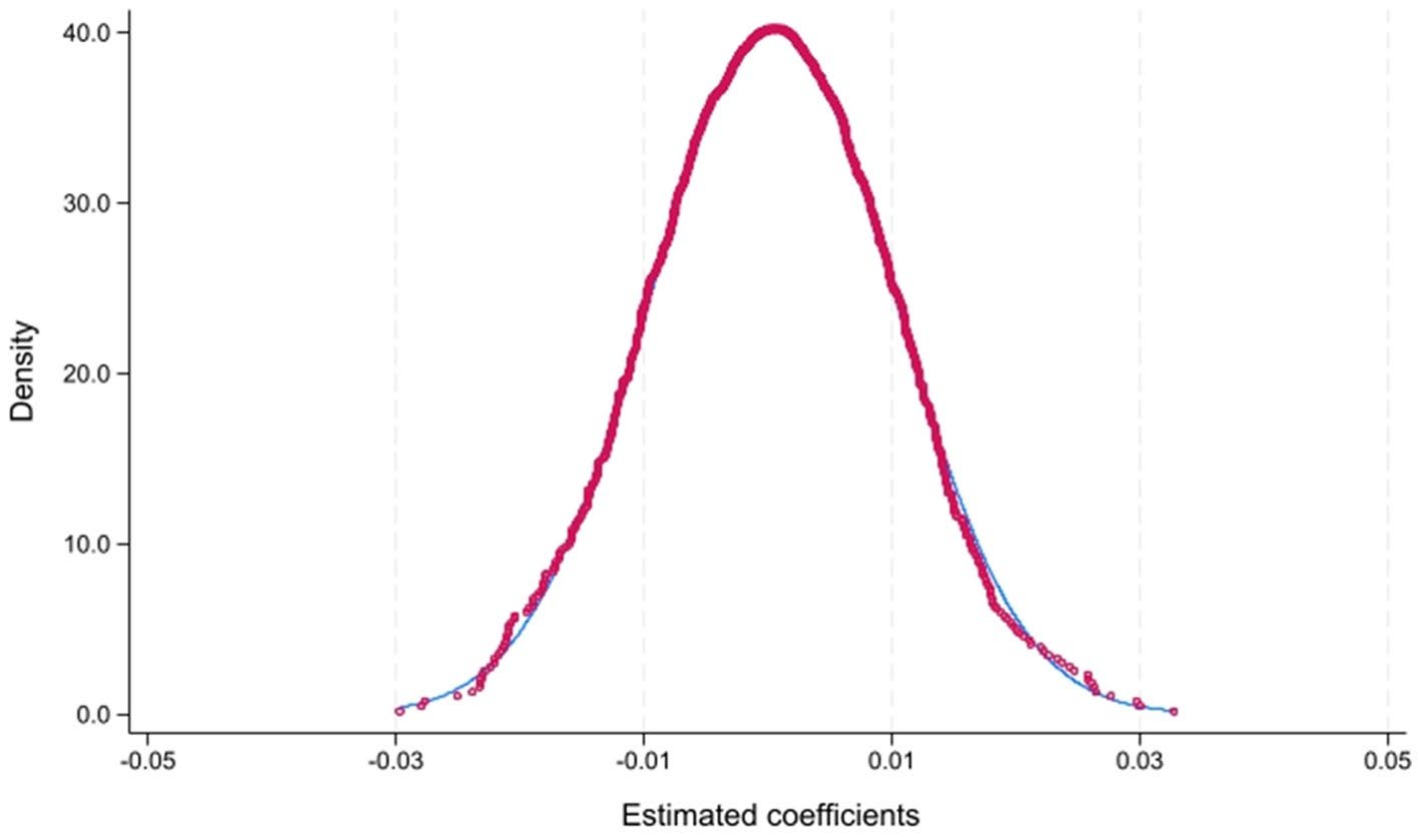
Placebo test.

To further confirm that the estimated LTCI effect is not driven by random factors or unobserved shocks, we conduct a placebo test as an additional robustness check. Specifically, we randomly assign cities to the treatment group and re-estimate the baseline specification 1,000 times. The simulated coefficient distribution clusters around zero and is statistically different from the actual estimate of 0.0860, which lies outside the simulated distribution (Figure 4). This evidence further supports the robustness of our baseline findings.

#### 5.2.6 Model averaging

As an additional robustness check, this subsection examines whether the results are sensitive to model uncertainty. Model averaging addresses model uncertainty by assigning weights to alternative specifications according to selected information criteria. In this study, we apply this approach and base the weighting process on four measures, AIC, BIC, AICC, and NOIC, to mitigate the potential influence of model uncertainty on the results. As reported in Table 6, the coefficient on the core explanatory variable remains significantly positive across all specifications, indicating that the LTCI policy continues to be robustly associated with higher regional housing prices even after accounting for model uncertainty.

**Table 6:** Model averaging results.

Variables	House_price			
	AIC	BIC	AICC	NOIC
	(1)	(2)	(3)	(4)
LTCI	0.0862*** (0.012)	0.0868*** (0.012)	0.0863*** (0.012)	0.2839* (0.202)
Control	Yes	Yes	Yes	Yes
City FE	Yes	Yes	Yes	Yes
Year FE	Yes	Yes	Yes	Yes
Observations	5,024	5,024	5,024	5,024
R-squared	0.948	0.948	0.948	0.948

***, **, and * denote statistical significance at the 1, 5, and 10% levels, respectively. Standard errors clustered at the city level are reported in parentheses.

#### 5.2.7 Excluding self-initiated pilot cities

The analysis next examines whether the estimated effect is subject to potential self-selection bias. The baseline regressions include both nationally designated pilot cities and self-initiated pilot cities. A potential concern is that self-initiated pilots may be more subject to self-selection bias. To address this, we restrict the sample to only the pilot cities officially designated by the MHRSS. As reported in Table 7, the main conclusions remain unchanged.

**Table 7:** Results excluding self-initiated pilot cities.

Variables	House_price	
	(1)	(2)
LTCI	0.0723** (0.030)	0.0803*** (0.027)
Control	No	Yes
City FE	Yes	Yes
Year FE	Yes	Yes
Observations	4,551	4,551
R-squared	0.936	0.939

***, **, and * denote statistical significance at the 1, 5, and 10% levels, respectively. Standard errors clustered at the city level are reported in parentheses. Column (1) reports the baseline two-way fixed effects (TWFE) regression without control variables, and Column (2) reports the specification including control variables. Both regressions are based on the subsample of nationally designated pilot cities.

## 6 Further analysis

### 6.1 Mechanism analysis

#### 6.1.1 Older adult migration

The mechanism through which LTCI influences the migration of older adults is primarily resource-based rather than welfare-based. Although LTCI does not automatically cover all older adult migrants, it promotes the optimization of care resource allocation in pilot cities, such as by expanding long-term care facilities, training caregivers, and integrating medical and care systems. These improvements enhance the perceived accessibility and quality of older adults care services, which in turn increase the attractiveness of pilot cities as retirement destinations and stimulate housing demand (32).

The data on the migration of older adults used in this study are obtained from Huang et al. (37), who estimate in-migration, out-migration, and net migration at the provincial level using the Sixth and Seventh National Population Censuses (2010 and 2020) as well as the 1% National Population Sample Surveys from 2005 and 2015. Since these data are not available on an annual basis, we follow Huang et al. and assign the provincial-level estimates to all prefecture-level cities within each province, extending each observation to cover the corresponding interval between survey years. This approach allows us to construct an unbalanced panel that matches the temporal span of the LTCI dataset. Using the specification in Equation 2, the regression results are reported in Columns (1)–(3) of Table 8. The findings show that LTCI significantly increases both the in-migration and net migration of older adults, but has no statistically significant effect on their out-migration. Overall, these results support Hypothesis 2: LTCI raises regional housing prices by attracting the in-migration of older adults and thereby increasing local housing demand.

**Table 8:** Mechanism analysis: older adults migration and real estate development.

Variables	Migration_in	Migration_out	Net_migration	lnRE_Completions	lnRE_Practitioners
	Older adult migration			Real estate investment	
	(1)	(2)	(3)	(4)	(5)
LTCI	1.8289*** (0.699)	0.1494 (0.314)	1.6795*** (0.628)	−0.1575** (0.064)	−0.0605 (0.058)
Control	Yes	Yes	Yes	Yes	Yes
City FE	Yes	Yes	Yes	Yes	Yes
Year FE	Yes	Yes	Yes	Yes	Yes
Observations	4,030	4,030	4,030	4,388	4,480
R-squared	0.830	0.874	0.836	0.920	0.896

***, **, and * denote significance at the 1, 5, and 10% levels, respectively. Standard errors clustered at the city level are reported in parentheses.

The above results confirm that LTCI promotes the in-migration of older adults. However, it should be noted that LTCI eligibility is institutionally linked to basic medical insurance enrollment, and in some pilot cities only urban employee participants are eligible for LTCI benefits. This institutional constraint implies that LTCI does not directly attract the migration of older adults through individual-level benefit eligibility. Instead, it enhances the overall provision of older adult care and healthcare resources, thereby improving the living environment and indirectly increasing the attractiveness of pilot cities to older population. To empirically examine this indirect mechanism, we consider two potential channels: healthcare resources and living environment. Specifically, we use the number of licensed doctors as a proxy for medical professionals and the number of hospitals as a proxy for medical infrastructure, and then re-estimate Equation 2. As shown in Columns (1)–(2) of Table 9, both variables are significantly and positively affected by LTCI at the 1% level, indicating that the policy fosters medical resource agglomeration, which is known to influence older adult migration (15). In addition, the living environment constitutes another important determinant of older adult migration (16). We use the total area of urban green parks as a proxy for environmental quality. Column (3) of Table 9 shows that LTCI significantly increases green park area, thereby enhancing the living environment and making cities more attractive to older adults.

**Table 9:** Mechanism analysis: healthcare resources and living environment.

Variables	lndoctor	lnhospital	Greenpark
	Medical resources		Living environment
	(1)	(2)	(3)
LTCI	0.0616*** (0.023)	0.2420*** (0.046)	0.1333*** (0.043)
Control	Yes	Yes	Yes
City FE	Yes	Yes	Yes
Year FE	Yes	Yes	Yes
Observations	5,014	5,020	4,999
R-squared	0.946	0.806	0.840

***, **, and * denote significance at the 1, 5, and 10% levels, respectively. Standard errors clustered at the city level are reported in parentheses.

#### 6.1.2 Real estate investment

Another potential mechanism through which LTCI could raise housing prices is by stimulating local real estate investment. By lowering household medical expenditures, LTCI may encourage a reallocation of resources toward non-healthcare sectors, such as real estate investment (14). In addition, the policy may enhance the appeal of housing designed for older adults as an investment sector, encouraging the development of retirement communities, senior apartments, and related facilities, which could contribute to higher housing prices.

To test this mechanism, we use real estate investment completions and the number of real estate practitioners as indicators, incorporating them into Model (2). The results, reported in Columns (4)–(5) of Table 8, show that LTCI has no statistically significant effect on the number of real estate practitioners, while exerting a significant negative effect on real estate investment completions. Overall, these findings do not support the hypothesis that LTCI stimulates real estate investment, and thus Hypothesis 3 is not confirmed.

### 6.2 Heterogeneity analysis

#### 6.2.1 Policy design of LTCI

##### 6.2.1.1 Service coverage

Most LTCI pilot cities reimburse both home-based care and institutional care services for older adults, whereas approximately 10% of pilot cities provide reimbursement only for institutional care. This arrangement may have a limited effect on housing prices, as older adults may choose institutional living rather than purchasing or renting new housing.

To examine whether the scope of service coverage affects the estimated impact, we divide the sample into two groups: those that reimburse both home-based care and institutional care, and those that reimburse only institutional care. We then estimate the effect for each subsample relative to the control group. As shown in columns (1) and (2) of Table 10, the estimated effect of LTCI on housing prices is significantly positive only in the home-based plus institutional care group. This pattern is consistent with the proposed migration-based mechanism, as expanded housing demand is more likely when home-based care is covered.

**Table 10:** Heterogeneity analysis: service coverage and pilot type.

Variables	House_price			
	Service coverage		Pilot type	
	Home + Institutional care	Institutional care only	Nationally designated pilots	Self-initiated pilots
	(1)	(2)	(3)	(4)
LTCI	0.1037*** (0.024)	−0.0565 (0.047)	0.0803*** (0.027)	0.1033** (0.040)
Control	Yes	Yes	Yes	Yes
City FE	Yes	Yes	Yes	Yes
Year FE	Yes	Yes	Yes	Yes
Observations	4,915	3,888	4,551	4,252
R-squared	0.945	0.932	0.939	0.941

***, **, and * denote statistical significance at the 1, 5, and 10% levels, respectively. Robust standard errors are clustered at the city level and reported in parentheses. Although the treatment cities in Columns (1) and (2), as well as Columns (3) and (4), are mutually exclusive, both regressions include the same set of non-pilot cities as the control group. Therefore, the total number of observations in the two columns combined exceeds the full sample size.

##### 6.2.1.2 Pilot type

In some regions with high social welfare demand and strong fiscal capacity, LTCI has been implemented independently at the local level, accounting for about 38% of pilot cities. We test whether the policy effects differ between these self-initiated pilots and those launched under central government directives, which offers insights for broader policy adoption.

Following the same approach, we distinguish between nationally designated pilot cities and self-initiated pilot cities, and estimate the effect for each subsample relative to the control group. This acts as an additional robustness check. As reported in columns (3) and (4) of Table 10, both types of pilots show a significant positive association between LTCI and housing prices. This suggests that differences in the implementing authority may not influence the impact of LTCI on housing prices, which reinforces the validity of the baseline findings.

#### 6.2.2 Regional context

##### 6.2.2.1 Geographic location

There is considerable inequality in the distribution of healthcare resources in China, with the eastern region concentrating the majority of these resources compared with the central and western regions (44). This spatial inequality may influence how LTCI affects regional housing prices, since areas with greater healthcare availability tend to experience stronger housing demand (24).

Following the classification standard of the National Bureau of Statistics of China, we divide the sample into Eastern cities and Non-Eastern cities. Columns (1) and (2) of Table 11 report the results. The effect of LTCI on housing prices is significantly positive at the 1% level in Eastern cities, while the effect is statistically insignificant in Non-Eastern cities. This finding reinforces the earlier conclusion that healthcare resources constitute an important driver of the in-migration of older adults, which ultimately generates spillover effects on housing costs.

**Table 11:** Heterogeneity analysis: geographic location and environmental quality.

Variables	House_price			
	Geographic location		Environmental quality	
	Eastern	Non-eastern	High emission	Low emission
	(1)	(2)	(3)	(4)
LTCI	0.1242*** (0.034)	0.0038 (0.033)	0.0439 (0.050)	0.1052*** (0.030)
Control	Yes	Yes	Yes	Yes
City FE	Yes	Yes	Yes	Yes
Year FE	Yes	Yes	Yes	Yes
Observations	1,783	3,241	2,627	2,385
R-squared	0.949	0.928	0.919	0.940

***, **, and * indicate statistical significance at the 1, 5, and 10% levels, respectively. Standard errors clustered at the city level are reported in parentheses.

##### 6.2.2.2 Environmental quality

Poor environmental conditions impose negative externalities, adversely affecting residents’ physical and mental health, especially among older adults (45, 46). Consequently, migration driven by the desire to avoid pollution may reduce local housing demand (19, 47).

We categorize the sample into high-emission and low-emission cities based on sulfur dioxide (SO₂) emissions per unit of GDP, dividing the cities at the 50th percentile (median) threshold. The results, reported in Columns (3)–(4) of Table 11, indicate the estimated effects. The positive effect of LTCI on housing prices is statistically significant only in low-emission cities, suggesting that environmental quality plays a meaningful role in shaping the policy’s housing market impact (48).

## 7 Discussion

The empirical results reveal that the implementation of China’s Long-Term Care Insurance (LTCI) program exerts a significant positive effect on regional housing prices, confirming the theoretical expectation that welfare policies can generate market spillovers through both population migration and household consumption channels. Within the framework of welfare economics and spatial population economics, LTCI improves the accessibility and quality of care services for older adults and reshapes the spatial distribution of welfare benefits, thereby enhancing the attractiveness of pilot cities and increasing local housing demand. Meanwhile, by reducing out-of-pocket medical expenditures and improving financial security, LTCI alleviates household liquidity constraints, which in turn stimulates real estate investment and housing consumption. Together, these mechanisms illustrate how health-related social policies can extend beyond their immediate welfare objectives to affect broader socioeconomic and spatial outcomes.

The pioneering contribution of this study lies in extending the literature on LTCI beyond its well-documented health benefits to encompass its indirect economic costs through the housing market. Previous studies have mainly emphasized the role of LTCI in promoting social equity, household welfare, and healthcare affordability, focusing on its effects on medical expenditures and the allocation of care resources (49–51). Building on this foundation, the present study explores its impact on housing prices—an important social dimension. By identifying the migration of older adults as a key mechanism, this study reveals how welfare-induced mobility contributes to spatially differentiated housing effects. Furthermore, by evaluating variations in policy effects across service coverage, geographic location, and environmental quality, the study bridges social policy research and urban economics, offering valuable insights for both welfare policy design and housing market regulation. These effects, however, are not spatially uniform. The heterogeneity analysis indicates that the price-enhancing effect of LTCI is significantly stronger in eastern regions, low-pollution cities, and cities offering home-based care services for older adults. These differentiated outcomes can be interpreted through the spatial equilibrium mechanism: regions with stronger economic capacity and better environmental quality exhibit higher welfare capitalization efficiency, meaning that the benefits of LTCI are more easily reflected in property values. In contrast, western or heavily polluted cities face lower population inflows and weaker market responses, suggesting that welfare policies alone cannot offset structural disadvantages in livability and infrastructure.

These findings carry several policy implications. First, to prevent overheating in high-demand housing markets, particularly in developed eastern cities, local governments should adopt measures to decouple welfare improvements from speculative housing demand—for instance, by increasing the supply of older-adult-friendly rental housing and strengthening market supervision. Second, to reduce welfare-induced spatial inequalities, the central government should consider harmonizing LTCI eligibility criteria and reimbursement standards across regions, thereby narrowing inter-city welfare differentials that drive selective migration of older adults. Third, in cities with weaker economic foundations, the government should prioritize capacity building in community- and home-based care services rather than large-scale institutional facilities, so as to improve welfare accessibility without generating excessive housing price pressure. Finally, policymakers should recognize that welfare policies and real estate markets are interdependent; sustainable expansion of LTCI should therefore be accompanied by spatial coordination mechanisms that balance welfare provision, housing affordability, and regional equity.

Despite the robustness of these findings, a number of limitations remain. First, due to data availability constraints, older adult migration is measured at the provincial rather than city level, whereas LTCI pilots are implemented at the city level. This mismatch in administrative scale may introduce measurement error and potentially attenuate the estimated effects. In particular, city-level variation in migration responses might be diluted when aggregated to the province level, leading to an underestimation of the local impact of LTCI. Future research could benefit from incorporating high-frequency or administrative mobility data, such as mobile phone tracking data, to better capture city-level migration dynamics.

Overall, the study contributes to the emerging literature on the spatial capitalization of welfare policies, showing that social insurance reforms not only improve individual well-being but also reshape urban spatial dynamics. However, the policy implications must be interpreted with caution. Since LTCI participation remains restricted mainly to urban employee medical insurance beneficiaries, the observed housing effects primarily reflect institutional rather than universal welfare expansion. Future policy adjustments that broaden coverage to rural and migrant older adults may alter both the magnitude and spatial pattern of these effects.

## 8 Conclusion

This study provides systematic evidence that China’s Long-Term Care Insurance (LTCI) program, while designed to enhance the welfare of older adults, has generated significant and uneven economic spillovers in the housing market. Using a staggered difference-in-differences model and panel data for 285 cities from 2003 to 2022, the analysis reveals that LTCI increases regional housing prices by approximately 8–9%, primarily through the in-migration of older adults rather than real estate investment. The findings highlight that welfare expansion, when implemented in spatially heterogeneous contexts, can reshape urban demand structures and exacerbate regional disparities.

Building on these findings, the paper advances the literature by integrating welfare economics with spatial population theory to explain how social policy interventions are capitalized into property markets. It demonstrates that the effects of LTCI are most pronounced in eastern, low-pollution, and home-based care cities, underscoring the importance of local capacity, environmental quality, and policy design in shaping welfare outcomes.

For policymakers, these insights call for the spatial coordination of welfare and housing systems. The sustainable expansion of LTCI should be accompanied by differentiated strategies—preventing housing overheating in developed regions while enhancing care capacity and environmental quality in lagging areas. Future research could employ micro-level or city-level migration and housing transaction data to more precisely identify the mechanisms through which LTCI affects local housing markets, and to capture intra-urban variations that remain unobservable in aggregate analysis.

## Data Availability

The original contributions presented in the study are included in the article/Supplementary material, further inquiries can be directed to the corresponding author.

## Footnotes

^1^ https://www.anjuke.com/

^2^ https://mohrss.gov.cn/
