# Supplementary material for: Unexpected costs: the impact of Long-Term Care Insurance on housing prices
Source: Front Public Health. 2025 Nov 13;13:1702221. doi: 10.3389/fpubh.2025.1702221 (PMC12657146; doi:10.3389/fpubh.2025.1702221)
Supplement: Supplementary file 1 [file Table_1.DOCX]

Supplementary Material

# Supplementary Data

Supplementary Table 1. List of LTCI Pilot Cities and Policy Design Features

| Province | City | Year of Implementation | Covered  Population | reimbursement method | service coverage | pilot type |
| --- | --- | --- | --- | --- | --- | --- |
|  |  |  | ①Ue= Urban employees  ②UeUr= Urban employees + Urban-rural residents | ①S= Services  ②CS= Cash + Services | ①Hi= Home + Institu-tional care  ②Ic= Institutional care | ①Fb= First batch  ②Sb= Second batch  ③Sip=Self-initiated pilots |
| Shandong | Qingdao | 2012 | UeUr | S | Hi | Fb |
| Shandong | Dongying | 2014 | UeUr | CS | Hi | Fb |
| Shandong | Weifang | 2014 | Ue | S | Hi | Fb |
| Shandong | Liaocheng | 2014 | Ue | S | Hi | Fb |
| Shandong | Rizhao | 2015 | Ue | S | Hi | Fb |
| Jilin | Changchun | 2015 | UeUr | S | Ic | Fb |
| Jiangsu | Nantong | 2015 | UeUr | S | Hi | Fb |
| Shandong | Jinan | 2016 | Ue | S | Hi | Fb |
| Jilin | Songyuan | 2016 | UeUr | S | Ic | Fb |
| Beijing | Beijing | 2016 | UeUr | S | Hi | Sip |
| Hebei | Xingtai | 2016 | UeUr | S | Hi | Sip |
| Jilin | Jilin City | 2016 | UeUr | S | Ic | Fb |
| Hubei | Jingmen | 2016 | UeUr | S | Hi | Fb |
| Hebei | Chengde | 2016 | Ue | S | Hi | Fb |
| Jiangxi | Shangrao | 2016 | Ue | CS | Hi | Fb |
| Shanghai | Shanghai | 2016 | UeUr | S | Hi | Fb |
| Anhui | Anqing | 2017 | Ue | CS | Hi | Fb |
| Zhejiang | Hangzhou | 2017 | UeUr | S | Hi | Sip |
| Jiangsu | Xuzhou | 2017 | UeUr | CS | Hi | Sip |
| Sichuan | Chengdu | 2017 | Ue | S | Hi | Fb |
| Xinjiang | Shihezi | 2017 | UeUr | CS | Hi | Fb |
| Jiangsu | Suzhou | 2017 | UeUr | S | Hi | Fb |
| Zhejiang | Jiaxing | 2017 | UeUr | S | Hi | Sip |
| Heilongjiang | Qiqihar | 2017 | Ue | S | Hi | Fb |
| Guangdong | Guangzhou | 2017 | Ue | S | Hi | Fb |
| Shandong | Linyi | 2017 | Ue | S | Hi | Fb |
| Jilin | Tonghua | 2017 | UeUr | S | Ic | Fb |
| Shanxi | Linfen | 2017 | UeUr | S | Hi | Sip |
| Zhejiang | Ningbo | 2017 | Ue | S | Ic | Fb |
| Shandong | Zibo | 2017 | Ue | S | Hi | Fb |
| Shandong | Tai'an | 2017 | Ue | S | Hi | Fb |
| Xinjiang | Changji | 2017 | Ue | CS | Hi | Sip |
| Chongqing | Chongqing | 2017 | Ue | S | Hi | Fb |
| Shandong | Binzhou | 2017 | Ue | CS | Hi | Fb |
| Jilin | Baishan | 2017 | UeUr | S | Ic | Fb |
| Xinjiang | Karamay | 2018 | UeUr | CS | Hi | Sip |
| Zhejiang | Taizhou | 2018 | UeUr | S | Hi | Sip |
| Shandong | Weihai | 2018 | Ue | S | Hi | Fb |
| Shandong | Heze | 2018 | Ue | S | Hi | Fb |
| Shandong | Yantai | 2018 | Ue | S | Hi | Fb |
| Shandong | Zaozhuang | 2018 | Ue | S | Hi | Fb |
| Hunan | Changsha | 2018 | Ue | S | Hi | Sip |
| Zhejiang | Jinhua | 2018 | UeUr | S | Hi | Sip |
| Jiangsu | Changzhou | 2018 | UeUr | S | Hi | Sip |
| Shandong | Jining | 2018 | Ue | S | Hi | Fb |
| Jiangsu | Yangzhou | 2018 | Ue | CS | Hi | Sip |
| Guangxi | Hezhou | 2018 | Ue | S | Hi | Sip |
| Xinjiang | Urumqi | 2018 | Ue | S | Hi | Sip |
| Shandong | Dezhou | 2018 | Ue | CS | Hi | Fb |
| Jiangsu | Wuxi | 2018 | UeUr | CS | Hi | Sip |
| Zhejiang | Zhoushan | 2019 | Ue | CS | Hi | Sip |
| Inner Mongolia | Manzhouli | 2019 | UeUr | S | Hi | Sip |
| Zhejiang | Wenzhou | 2019 | Ue | CS | Hi | Sip |
| Jiangsu | Taizhou | 2019 | UeUr | S | Hi | Sip |
| Hebei | Qinhuangdao | 2019 | Ue | S | Hi | Sip |
| Jilin | Yanbian | 2019 | Ue | S | Hi | Fb |
| Inner Mongolia | Wuhai | 2019 | UeUr | S | Hi | Sip |
| Hebei | Tangshan | 2019 | UeUr | CS | Hi | Sip |
| Hebei | Shijiazhuang | 2019 | UeUr | CS | Hi | Sip |
| Hubei | Tianmen | 2019 | Ue | S | Hi | Sip |
| Hubei | Yichang | 2019 | Ue | S | Hi | Sip |
| Hebei | Baoding | 2020 | Ue | S | Hi | Sip |
| Gansu | Gannan Tibetan Autonomous Prefecture | 2020 | Ue | S | Hi | Sb |
| Fujian | Quanzhou | 2020 | UeUr | S | Hi | Sip |
| Shaanxi | Hanzhong | 2020 | Ue | CS | Hi | Sb |
| Guizhou | Qianxinan Prefecture | 2020 | Ue | CS | Hi | Sb |
| Guangdong | Shenzhen | 2020 | UeUr | S | Hi | Sip |
| Hunan | Xiangtan | 2020 | Ue | S | Hi | Sb |
| Liaoning | Panjin | 2020 | Ue | S | Hi | Sb |
| Fujian | Fuzhou | 2020 | Ue | S | Hi | Sb |
| Yunnan | Kunming | 2020 | Ue | S | Hi | Sb |
| Henan | Kaifeng | 2020 | Ue | CS | Hi | Sb |
| Inner Mongolia | Hohhot | 2020 | UeUr | S | Hi | Sb |
| Guangxi | Nanning | 2021 | Ue | S | Hi | Sb |
| Tianjin | Tianjin | 2021 | Ue | S | Hi | Sb |
| Shanxi | Jincheng | 2021 | Ue | CS | Hi | Sb |

Notes: Due to data limitations, the underlined cities are outside the sample study.

Supplementary Table 2. Balance test of the nearest neighbor matching

| Variables | Unmatched | Mean | | Bias (%) | Reduce  bias (%) | T-test | |
| --- | --- | --- | --- | --- | --- | --- | --- |
|  | Matched | Treated | Control |  |  | T | P>T |
| indus | U | 48.949 | 48.003 | 7.7 |  | 2.32 | 0.020 |
|  | M | 49.038 | 49.252 | -1.7 | 77.4 | -0.46 | 0.646 |
| lnperGDP | U | 10.948 | 10.447 | 66.6 |  | 19.54 | 0.000 |
|  | M | 10.944 | 10.946 | -0.3 | 99.6 | -0.07 | 0.941 |
| lnpop | U | 5.0696 | 4.4780 | 75.5 |  | 24.61 | 0.000 |
|  | M | 5.0576 | 5.0396 | 2.3 | 97.0 | 0.54 | 0.593 |
| edu | U | 0.0201 | 0.0253 | -43.4 |  | -12.25 | 0.000 |
|  | M | 0.0201 | 0.0198 | 2.8 | 93.4 | 0.92 | 0.358 |
| fis | U | 0.3186 | 0.5566 | -58.2 |  | -15.62 | 0.000 |
|  | M | 0.3192 | 0.3145 | 1.2 | 98.0 | 0.44 | 0.661 |
| so2 | U | 1.4231 | 2.4740 | -28.1 |  | -7.51 | 0.000 |
|  | M | 1.4299 | 1.5086 | -2.1 | 92.5 | -0.51 | 0.612 |

Notes: In the second column, U refers to the sample before employing the PSM, while M denotes the matched sample after applying the PSM.

Supplementary Table 3. Balance test of the radius matching

| Variables | Unmatched | Mean | | Bias (%) | Reduce  bias (%) | T-test | |
| --- | --- | --- | --- | --- | --- | --- | --- |
|  | Matched | Treated | Control |  |  | T | P>T |
| indus | U | 48.949 | 48.003 | 7.7 |  | 2.32 | 0.020 |
|  | M | 49.038 | 49.273 | -1.9 | 75.2 | -0.50 | 0.616 |
| lnperGDP | U | 10.948 | 10.447 | 66.6 |  | 19.54 | 0.000 |
|  | M | 10.944 | 10.944 | 0.1 | 99.9 | 0.01 | 0.989 |
| lnpop | U | 5.0696 | 4.4780 | 75.5 |  | 24.61 | 0.000 |
|  | M | 5.0576 | 5.0468 | 1.4 | 98.2 | 0.32 | 0.747 |
| edu | U | 0.0201 | 0.0253 | -43.4 |  | -12.25 | 0.000 |
|  | M | 0.0201 | 0.0199 | 2.0 | 95.3 | 0.65 | 0.515 |
| fis | U | 0.3186 | 0.5566 | -58.2 |  | -15.62 | 0.000 |
|  | M | 0.3192 | 0.3163 | 0.7 | 98.8 | 0.28 | 0.783 |
| so2 | U | 1.4231 | 2.4740 | -28.1 |  | -7.51 | 0.000 |
|  | M | 1.4299 | 1.4577 | -0.7 | 97.4 | -0.19 | 0.853 |

Notes: In the second column, U refers to the sample before employing the PSM, while M denotes the matched sample after applying the PSM.

Supplementary Table 4. Balance test of the kernel matching

| Variables | Unmatched | Mean | | Bias (%) | Reduce  bias (%) | T-test | |
| --- | --- | --- | --- | --- | --- | --- | --- |
|  | Matched | Treated | Control |  |  | T | P>T |
| indus | U | 48.949 | 48.003 | 7.7 |  | 2.32 | 0.020 |
|  | M | 49.038 | 49.415 | -3.1 | 60.2 | -0.80 | 0.424 |
| lnperGDP | U | 10.948 | 10.447 | 66.6 |  | 19.54 | 0.000 |
|  | M | 10.944 | 10.936 | 1.1 | 98.4 | 0.29 | 0.774 |
| lnpop | U | 5.0696 | 4.4780 | 75.5 |  | 24.61 | 0.000 |
|  | M | 5.0576 | 5.0285 | 3.7 | 95.1 | 0.87 | 0.385 |
| edu | U | 0.0201 | 0.0253 | -43.4 |  | -12.25 | 0.000 |
|  | M | 0.0201 | 0.0199 | 1.8 | 95.7 | 0.59 | 0.558 |
| fis | U | 0.3186 | 0.5566 | -58.2 |  | -15.62 | 0.000 |
|  | M | 0.3192 | 0.3201 | -0.2 | 99.6 | -0.08 | 0.933 |
| so2 | U | 1.4231 | 2.4740 | -28.1 |  | -7.51 | 0.000 |
|  | M | 1.4299 | 1.4866 | -1.5 | 94.6 | -0.37 | 0.708 |

Notes: In the second column, U refers to the sample before employing the PSM, while M denotes the matched sample after applying the PSM.

# Supplementary Figures and Tables

## Supplementary Figures

**
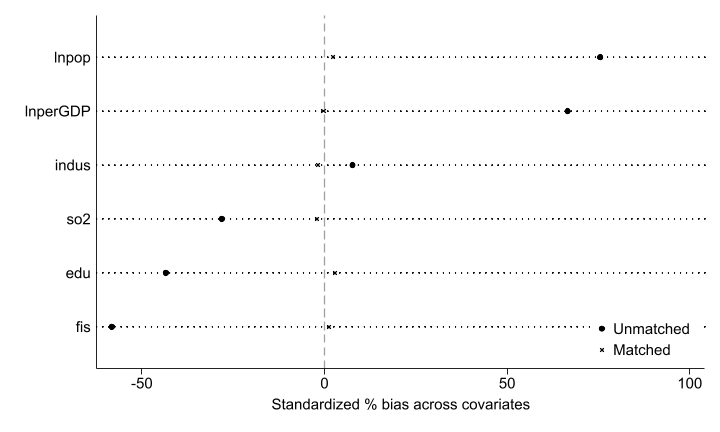
**

**Supplementary Figure 1.** Distribution of variables before and after nearest neighbor matching

**
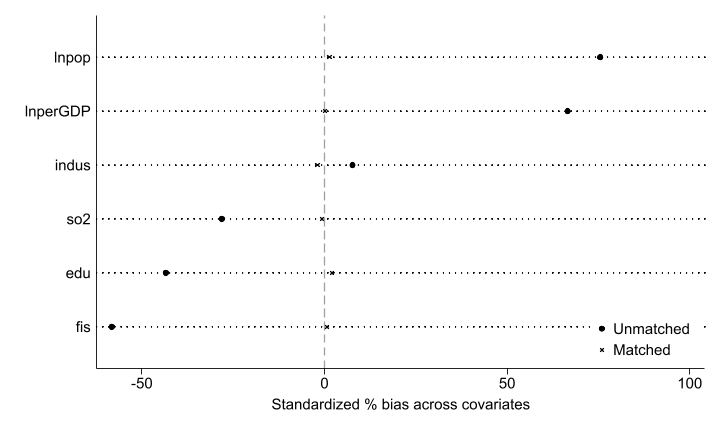
**

Supplementary Figure 2. Distribution of variables before and after radius matching

**
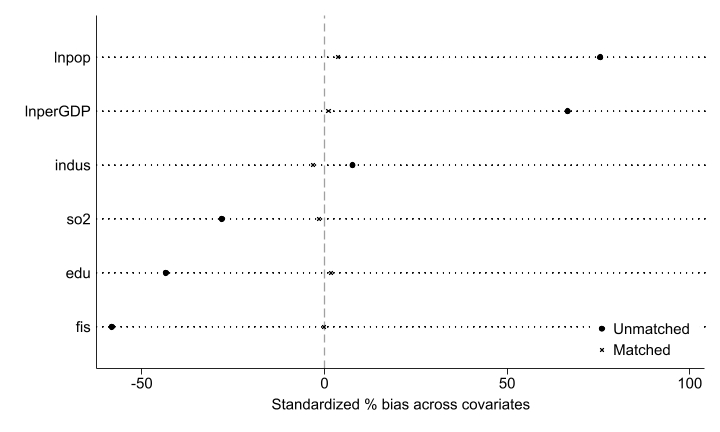
**

Supplementary Figure 3. Distribution of variables before and after kernel matching

**
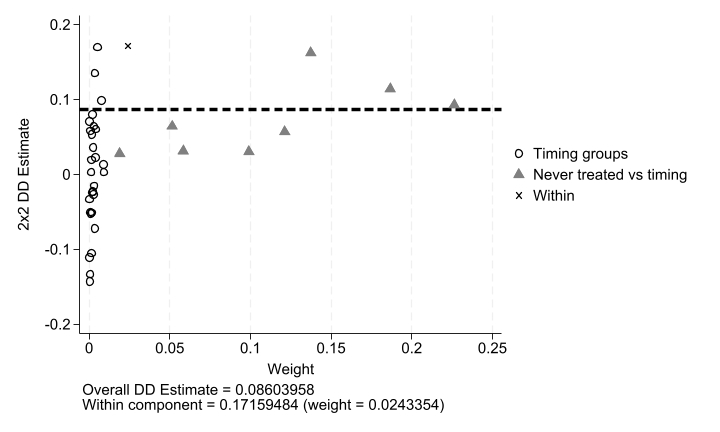
**

Supplementary Figure 4. Result of Goodman-Bacon decomposition
